# Cost-effectiveness of pemigatinib as a second-line treatment for advanced intrahepatic cholangiocarcinoma with fibroblast growth factor receptor 2 fusions in Taiwan: from the evidence of the phase II trial and the perspective of Taiwan's National Health Insurance Administration

**DOI:** 10.1186/s12962-023-00473-5

**Published:** 2023-09-11

**Authors:** Zi-Rong Chen, Chen-Han Chueh, Nai-Jung Chiang, Yi-Wen Tsai

**Affiliations:** 1 Institute of Health and Welfare Policy, College of Medicine, National Yang Ming Chiao Tung University, Taipei, Taiwan; 2 Department of Oncology, Taipei Veterans General Hospital, Taipei, Taiwan; 3 School of Medicine, College of Medicine, National Yang Ming Chiao Tung University, Taipei, Taiwan

**Keywords:** Pemigatinib, Intrahepataic cholangiocarcinoma, Targeted therapy, Economic evaluation, Cost-effectiveness analysis, Price listing strategy

## Abstract

**Background:**

In December 2022, the Taiwan National Health Insurance Administration (NHIA) announced the reimbursement of three dosages of pemigatinib 4.5 mg, 9 mg, and 13.5 mg for treating advanced intrahepatic cholangiocarcinoma (ICC) with fibroblast growth factor receptor 2 (*FGFR2*) fusions/rearrangements and set the reimbursement price for pemigatinib 4.5 mg at NT$6600. This study aims to analyze the cost-effectiveness of pemigatinib 13.5 mg as a second-line treatment compared to mFOLFOX and 5-FU chemotherapy for advanced ICC patients with *FGFR2* fusions/rearrangements from the perspective of Taiwan’s NHIA.

**Methods:**

This study used a 3-state partitioned survival model to analyze the 5 year cost-effectiveness of pemigatinib as a second-line treatment for advanced ICC patients in whom first-line gemcitabine-based chemotherapy failed and to compare the results with those for the mFOLFOX and 5-FU chemotherapy regimens. Overall survival and progression-free survival were estimated from the FIGHT-202 trial (pemigatinib), ABC-06 trial (mFOLFOX), and NIFTY trial (5-FU). The price of pemigatinib 13.5 mg was set at the potentially highest listing price (NT$17,820). Other parameters of utility, disutility, and costs related to advanced ICC were obtained from the published literature. The willingness-to-pay threshold was three times the forecasted gross domestic product per capita in 2022 (NT$2,928,570). A 3% discount rate was applied to quality-adjusted life-years (QALYs) and costs. Several scenario analyses were performed, including a gradual price reduction for pemigatinib. Deterministic sensitivity analysis, probabilistic sensitivity analysis (PSA), and value of information were performed to assess uncertainty.

**Results:**

Pemigatinib was not cost-effective compared to mFOLFOX or 5-FU in the base-case analysis. When the price of pemigatinib was reduced by 50% or more, pemigatinib gained a positive net monetary benefit (mFOLFOX: NT$55,374; 5-FU: NT$92,437) and a 72% (mFOLFOX) and 77.1% (5-FU) probability of being cost-effective. Most of the uncertainty came from the medication cost of pemigatinib, health state utility, and the overall survival associated with pemigatinib.

**Conclusions:**

According to the NCCN guidelines, the daily use of pemigatinib 13.5 mg at the hypothesized NHIA price of NT$17,820/13.5 mg was not cost-effective compared to mFOLFOX or 5-FU. The price reduction scenario suggested a 50% price reduction, NT$8910 per 13.5 mg, for advanced ICC patients with *FGFR2* fusions/rearrangements.

## Plain language summary

This study performed a cost-effectiveness analysis on the use of targeted therapy pemigatinib 13.5 mg daily in second-line treatment for Taiwanese patients with intrahepatic cholangiocarcinoma (ICC) harboring *FGFR2* fusions/rearrangements. This regimen was approved by the U.S. Food and Drug Administration in 2020 and recommended by the National Comprehensive Cancer Network (NCCN). Taiwan's National Health Insurance Administration (NHIA) has announced the reimbursement of three pemigatinib dosages of 4.5 mg, 9 mg, and 13.5 mg to be listed in the NHI coverage in 2022. However, as of the middle of April 2023, only the listing price for pemigatinib 4.5 mg has been determined, while pricing for the other two dosages remains pending. Based on a hypothesized NHIA price of NT$17,820/13.5 mg, this study evaluated the cost-effectiveness of pemigatinib 13.5 mg as a second-line treatment for advanced ICC with *FGFR2* fusions/rearrangements compared to mFOLFOX (a regimen recommended by NCCN) and 5-FU (a regimen fully covered by Taiwan NHIA) and recommended a listing price for NHIA as reference. Our study showed that the hypothesized price of NT$17,820/13.5 mg was not cost-effective compared to mFOLFOX or 5-FU. The price reduction scenario suggested a 50% reduction (NT$8910) in the hypothesized NHIA price for advanced ICC patients with *FGFR2* fusions/rearrangements.

## Key points

- Pemigatinib was associated with a positive INMB when the medication price was reduced by 50% to NT$8910 per pemigatinib 13.5 mg.
- Most of the uncertainty came from the medication cost of pemigatinib, utilities, and the overall survival associated with pemigatinib treatment.

## Background

Intrahepatic cholangiocarcinoma (ICC) is subtype of cholangiocarcinoma (CCA) and a rare cancer with an incidence of fewer than 6/100,000 people in most countries [1, 2]. Fusions or rearrangements of fibroblast growth factor receptor 2 (*FGFR2*) in CCA, particularly ICC, have been identified with the incidence of 10–25% [3]. In 2020, the U.S. Food and Drug Administration (FDA) approved the FGFR kinase inhibitor,pemigatinib, as a second-line treatment for advanced CCA patients with *FGFR2* fusions/rearrangements based on a single-arm phase II trial (FIGHT-202) [4, 5], followed by the approval of infigratinib in 2021 and futibatinib in 2022 [6, 7]. The National Comprehensive Cancer Network (NCCN) recommends second-line treatment with FGFR kinase inhibitors for ICC patients with *FGFR2* fusions/rearrangements and modified FOLFOX (mFOLFOX: a combination of oxaliplatin, folinic acid, and fluorouracil) for those without *FGFR2* alterations [8]. Currently, only pemigatinib has been approved by the Taiwan FDA for the aforementioned indications [9]. Modified FOLFOX, the NCCN-recommended second-line systemic therapy for advanced ICC, is not fully covered by Taiwan’s National Health Insurance (NHI). Instead, fluorouracil (5-FU) with leucovorin chemotherapy is the most commonly used regimen covered by the NHI.

Although the U.S. FDA accelerated the approvals of FGFR kinase inhibitors [4, 6, 7], high-priced pemigatinib has not been widely used or covered by third parties. Furthermore, the cost-effectiveness results of the National Institute for Health and Care Excellence (NICE) [10] and the Canada’s Drug and Health Technology Agency (CADTH) [11] were inconsistent. In Taiwan, the biomarker-driven regimen of pemigatinib and mFOLFOX recommended by the NCCN was not shown to be cost-effective compared to the current 5-FU regimen based on the pemigatinib unit price of NT$14,285, unless there was a 40% reduction in the proposed price from the manufacturer [12].

In October 2022, Taiwan's National Health Insurance Administration (NHIA) [13] announced the reimbursement of pemigatinib 4.5 mg and 9 mg with an expected unit price of NT$6600 for pemigatinib 4.5 mg. Two months later, the NHI covered pemigatinib 13.5 mg, but the listing price is currently being discussed [13]. This study aims to evaluate the cost-effectiveness of pemigatinib 13.5 mg per day as a second-line treatment and provide cost-effective pricing for advanced ICC with *FGFR2* fusions/rearrangements compared to mFOLFOX and 5-FU from the perspective of Taiwan's NHIA by using real-world NHI claims data based on a hypothesized NHIA price for pemigatinib 13.5 mg.

## Methods

### Cost-effectiveness analytical framework

**Fig. 1:**
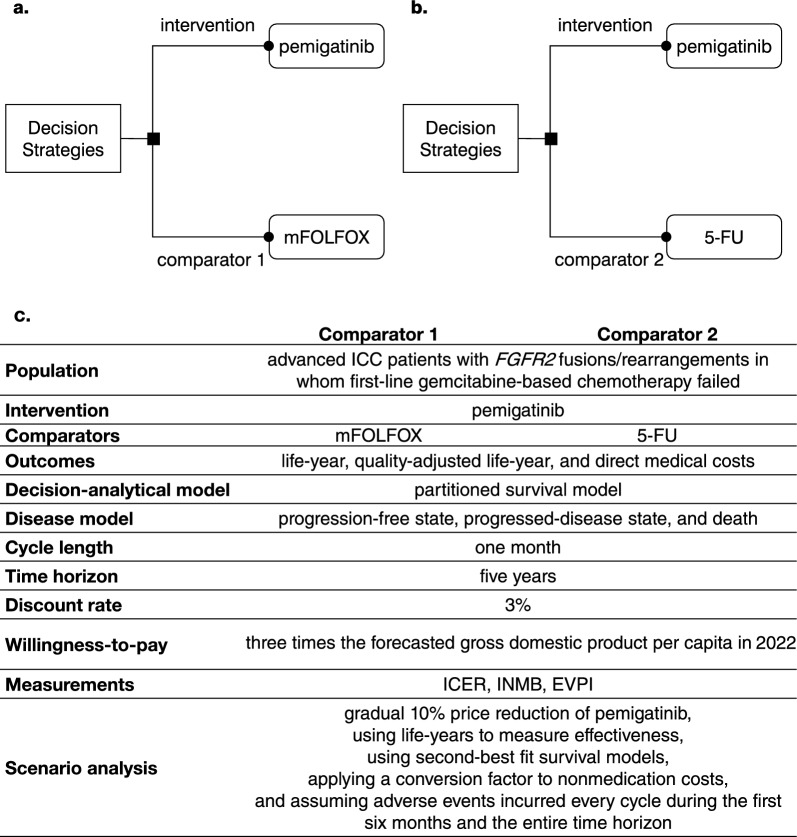
Decision strategies: intervention regimen provides pemigatinib 13.5 mg per day, comparator 1 provides mFOLFOX **a**, comparator 2 provides 5-FU **b**, and the cost-effectiveness analysis framework **c**. *ICC* intrahepatic cholangiocarcinoma, *FGFR2* fibroblast growth factor receptor 2, *mFOLFOX* a combination of oxaliplatin, folinic acid, and fluorouracil, *5-FU* fluorouracil, *ICER* incremental cost-effectiveness ratio, *INMB* incremental net monetary benefit, *EVPI* expected value of perfect information

This study aims to evaluate the cost-effectiveness of NHIA’s new reimbursement of pemigatinib 13.5 mg as a second-line treatment compared to mFOLFOX (Fig. 1a) and 5-FU (Fig. 1b) for advanced ICC patients with *FGFR2* fusions/rearrangements from the perspective of Taiwan’s NHIA.

This study was an update of our previous study, which evaluated the cost-effectiveness of NCCN-recommended treatment regimens [12]. The methodology and parameters were described in that previous study [12]. This study was reported following the Consolidated Health Economic Evaluation Reporting Standards [14, 15]. The cost-effectiveness analysis (CEA) framework is summarized in Fig. 1c.

### Clinical information on the target population

The target patients in this study were the study subjects of three trials, namely, FIGHT-202 (pemigatinib) [5], ABC-06 (mFOLFOX) [16], and NIFTY (5-FU) [17]. The inclusion criteria for these trials were adults with histologically or cytologically confirmed disease progression after at least one systemic cancer therapy (previous treatment with selective FGFR inhibitors was not allowed in FIGHT-202) [5, 16, 17]. The three trials differed in the inclusion criteria for age (NIFTY: aged ≥ 19 years; FIGHT-202 and ABC-06: aged ≥ 18 years) and Eastern Cooperative Oncology Group performance status (NIFTY and ABC-06: 0–1; FIGHT-202: 0–2) [5, 16, 17].

### Intervention and comparator treatment protocols

According to the published study [12], the current study assumed that the treatment regimens followed the treatment protocols of the three trials. In the intervention regimen, patients take 13.5 mg of oral pemigatinib every 3 weeks (2 weeks on and 1 week off) until disease progression [5]. In the first comparator regimen, patients receive oxaliplatin 85 mg/m^2^, L-folinic acid 175 mg (or folinic acid 350 mg), fluorouracil 400 mg/m^2^ (bolus), and fluorouracil 2400 mg/m^2^ as a 46 h continuous intravenous infusion every two weeks, as in the ABC-06 trial [16]. In the second comparator regimen, patients receive leucovorin 400 mg/m^2^ and fluorouracil 2400 mg/m^2^ every 2 weeks, as in the NIFTY trial [17]. In our study, we assumed that second-line systemic treatments would be provided up to the disease-progression state. When the disease progressed, patients would receive the same supportive care.

### Decision-analytical model and model inputs

We defined three mutually exclusive health states for economic evaluation: the progression-free (PF) state, the progressed-disease (PD) state, and death. Partitioned survival models were used to estimate the proportion of each health state membership in each cycle using the progression-free survival (PFS) and overall survival (OS) curves from the three trials: FIGHT-202, ABC-06, and NIFTY [18, 19]. The PFS and OS of the trials were obtained using a hybrid method: Kaplan‒Meier curves were used for the duration of the trials, and parametric distributions were used in the extrapolation period. The PFS and OS for pemigatinib had a log-normal and Weibull distribution, respectively; those for mFOLFOX were both log-normal distributions; and those for 5-FU had a generalized gamma and log-normal distribution, respectively [5, 12, 16, 17].

The model parameters are listed in Table 1. Genetic testing fees, medication costs for second-line therapy and nonmedication expenses (including all expenditures the NHIA paid, such as diagnostic fees, medication service fees, administration fees, and infusion-related fees) were included for the PF state; expenditure for supportive care was included for the PD state. The genetic testing fee was set at the market price and assumed to be uniform distribution. The hypothesized medication cost of pemigatinib (NT$6600 × 3 × 0.9 = NT$17,820) was set at the potential highest listing price and assumed to be a uniform distribution [13]. All other costs were derived from our previous study, which estimated the costs from NHI claims data and assumed gamma distributions [12]. For the PF state, the average costs per cycle of medication and other medical expenditures were NT$345,600 and NT$23,949 for pemigatinib users, NT$33,847 and NT$70,389 for mFOLFOX users, and NT$14,600 and NT$70,389 for 5-FU users, respectively. For the PD state, the average cost per cycle of supportive care was NT$37,518 for all patients [12].

**Table 1:** Model parameters, baseline values, ranges, and distributions for sensitivity analyses

Parameters and distributions	Estimated value	DSA		PSA	Source
		Range (± 25%)		Distribution	
1. Overall survival					
Pemigatinib: Weibull	Scale: 22.065 Shape: 1.536	17.486 1.184	27.843 1.993	Normal (22.065, 2.619) Normal (1.536, 0.204)	(5, 12)
mFOLFOX: log-normal	Intercept: 1.834 Scale: 0.873	1.642 0.742	2.023 1.027	Normal (1.834, 0.098) Normal (0.873, 0.072)	(12, 16)
5-FU: log-normal	Intercept: 1.764 Scale: 0.816	1.586 0.691	1.943 0.965	Normal (1.764, 0.091) Normal (0.816, 0.07)	(12, 17)
2. Progression-free survival					
Pemigatinib: log-normal	Intercept: 1.962 Scale: 0.971	1.76 0.82	2.164 1.15	Normal (1.962, 0.103) Normal (0.971, 0.084)	(5, 12)
mFOLFOX: log-normal	Intercept: 1.430 Scale: 0.754	1.265 0.643	1.595 0.885	Normal (1.43, 0.084) Normal (0.754, 0.062)	(12, 16)
5-FU: log-normal	Intercept: 0.8 Scale: 1.067	0.57 0.91	1.031 1.251	Normal (0.8, 0.118) Normal (1.067, 0.087)	(12, 17)
3. Genetic testing fee (NT$)	30,000	22,500	37,500	Uniform (22,500, 37,500)	Market price
4. Medication cost (per year, NT$)					
Pemigatinib	4,336,200	3,252,150	5,420,250	Uniform (3,252,150, 5,420,250)	(13)
mFOLFOX	412,087	309,065	515,108	Gamma (412,087, 617,747)	(12)
5-FU	177,759	133,320	222,199	Gamma (177,759, 282,466)	(12)
5. Nonmedication cost (per year, NT$)					
Pemigatinib	291,576	218,682	364,470	Gamma (291,576, 691,057)	(12)
Chemotherapy	856,986	642,740	1,071,233	Gamma (856,986, 1,067,455)	(12)
6. Supportive care cost (per year, NT$)	497,710	373,283	622,138	Gamma (497,710, 576,562)	(12)
7. Utility					
Progression-free state	0.760	0.57	0.95	Beta (4.7, 1.5)	(21)
Postprogression state	0.680	0.51	0.85	Beta (29, 13.6)	(21)
8. Disutility					
Intravenous therapy	0.025	0.0188	0.0313		(10, 20)
Grade 3 and higher AEs	0.160	0.1200	0.2000	Beta (36, 193)	(22)
9. Discount rate (per year)	0.03	0.0000	0.0500		(28)
10. Conversion factor	0.9			Uniform (0.8, 1)	

Costs listed in 2022 Taiwan dollars

*5-FU fluorouracil, AE adverse event, DSA* deterministic sensitivity analysis, *FGFR2* fibroblast growth factor receptor 2, *mFOLFOX* a combination of oxaliplatin, folinic acid, and fluorouracil, *PSA* probabilistic sensitivity analysis

As there is currently no evidence of utility or disutility due to adverse events for advanced ICC patients, this study used the same approach as NICE technology appraisal guidance [20, 21]. Health state utility values for the PF state and PD state were 0.76 and 0.68, respectively, and were assumed to be beta distributions [21]. Disutility due to adverse events was 0.16 [2, 22]; disutility in patients who received subcutaneous or intravenous therapies (comparators) was 0.025 [10, 20]. All adverse events were assumed to be incurred during the first cycle due to the lack of this information for advanced ICC patients [10, 20]. To be conservative, the proportion of any adverse events ≥ grade 3 was the sum of the proportions of each adverse event grade 3 in each trial. The proportions of adverse events ≥ grade 3 for each arm were 56%, 54%, and 18% for pemigatinib, mFOLFOX, and 5-FU, respectively [5, 16, 17].

### Cost-effectiveness analyses

In our study, the incremental cost-effectiveness ratio (ICER) was calculated as the incremental cost per additional quality-adjusted life-year (QALY) between pemigatinib and mFOLFOX (comparator 1) or the 5-FU treatment (comparator 2) in Taiwan. The incremental net monetary benefit (INMB) was calculated as the net monetary benefit of pemigatinib over mFOLFOX or 5-FU. An annual discount of 3% was applied to health outcomes and costs. Because there is no official willingness-to-pay (WTP) threshold in Taiwan, the WTP threshold was set as three times the forecasted gross domestic product (GDP) per capita in 2022 (NT$2,928,570) recommended by the WHO [23–25].

To evaluate the robustness of the base-case result, we performed a deterministic sensitivity analysis (DSA), probabilistic sensitivity analysis (PSA), and scenario analysis. In the DSA, the time horizon and the discount rate were set at a range of 3–15 years and 0–5%, respectively; parameters of the survival functions were set between the 95% confidence interval of the estimated value; the rest of the parameters were set between a ± 25% interval of the parameter’s estimated value. In the PSA, a Monte Carlo simulation of 1000 iterations was generated by sampling the parameters from specified distributions (Table 1). The cost-effectiveness acceptability curves and the expected value of perfect information (EVPI) were performed using the PSA results to better understand the uncertainty of our analysis. Several scenarios were performed in the scenario analyses: gradual 10% price reduction of pemigatinib, using life-years to measure effectiveness, using second-best fitting survival models, applying a conversion factor to nonmedication costs, and assuming adverse events incurred every cycle during the first six months and the entire time horizon.

### Model validation

The model validation followed the Assessment of the Validation Status of Health-Economic decision models [26]. Our conceptual model followed the same approach adopted by NICE and CADTH [10, 11]. We were unable to distinguish the mortalities attributed to the progressed state and the PF state due to the lack of individual-level data in the aforementioned trials. Therefore, a comparative model evaluation of the partitioned survival model and the Markov model could not be performed. To avoid errors, two different researchers on our team reviewed the decision-analytical model and programs.

## Results

### Base-case analysis

Pemigatinib was associated with a 0.59 QALY increase with incremental costs of NT$3,428,442 and a 0.68 QALY increase with incremental costs of NT$3,653,100, yielding an ICER of NT$5,814,700 per QALY and an ICER of NT$5,380,241 per QALY compared to mFOLFOX and 5-FU, respectively (Table 2).

**Table 2:** CEA results of base-case

Treatment strategy	Outcomes of Partitioned Survival Models			Incremental Changes	
	Intervention: Pemigatinib	Comparator 1: mFOLFOX	Comparator 2: 5-FU	Pemigatinib vs. mFOLFOX	Pemigatinib vs 5-FU
Cost	4,177,572	749,130	524,472	3,428,442	3,653,100
Total cost of PF state	3,780,469	577,970	369,229	3,202,499	3,411,240
Medication cost	3,514,168	187,675	63,430	3,326,493	3,450,738
Nonmedication cost	276,301	390,294	305,799	− 123,993	− 39,498
Total cost of PD state	397,103	171,160	155,243	225,943	241,860
LY					
PF state	0.81	0.46	0.36	0.35	0.45
Overall	1.61	0.80	0.67	0.81	0.94
QALY					
PF state	0.61	0.33	0.26	0.28	0.35
Overall	1.15	0.56	0.47	0.59	0.68
ICER					
Incremental cost per LY gained				4,238,063	3,888,175
Incremental cost per QALY gained				5,814,700	5,380,241
INMB					
LY				− 1,059,333	− 901,589
QALY				− 1,701,710	− 1,664,647
EVPI/person				43,139	23,608

Costs listed in 2022 Taiwan dollars

*EVPI expected value of perfect information, ICER incremental cost-effectiveness ratio, INMB* incremental net monetary benefit, *LY* life-year, *PD* progressed disease, *PF* progression free, *QALY* quality-adjusted life-year

### Base-case sensitivity analysis

**Fig. 2:**
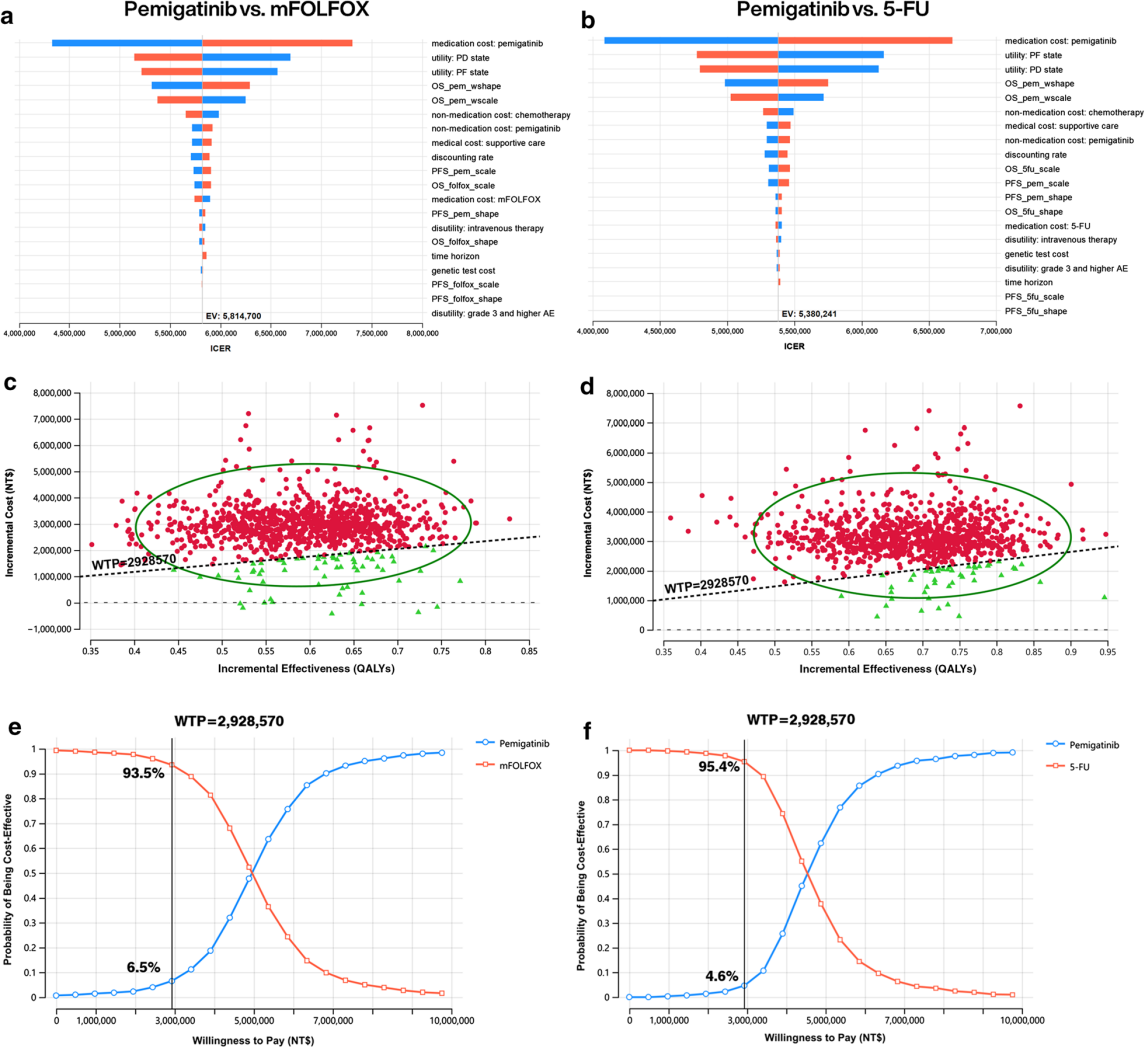
Deterministic sensitivity analysis and probabilistic sensitivity analysis results: Drivers of incremental cost per quality-adjusted life-year (QALY) gained for pemigatinib versus mFOLFOX **a** and 5-FU **b**. Incremental cost-effectiveness plane for pemigatinib versus mFOLFOX **c** and 5-FU **d**, and cost-effectiveness acceptance curve for pemigatinib versus mFOLFOX **e** and 5-FU **f**. Costs listed in 2022 NT dollars. The black lines indicate the willingness-to-pay threshold of NT$2,928,570 per QALY gained. *WTP* willingness to pay; *EV* expected value; *ICER* incremental cost-effectiveness ratio; *mFOLFOX* a combination of oxaliplatin, folinic acid, and fluorouracil; *5-FU* fluorouracil; *PD* progressed disease, *PF* progression free; *OS_pem_wshape* shape parameter of overall survival (pemigatinib), *OS_pem_wscale* scale parameter of overall survival (pemigatinib), *PFS_pem_scale* scale parameter of progression-free survival (pemigatinib), *OS_folfox_scale* scale parameter of overall survival (mFOLFOX), *PFS_pem_shape* shape parameter of progression-free survival (pemigatinib), *OS_folfox_shape* shape parameter of overall survival (mFOLFOX), *PFS_folfox_scale* scale parameter of progression-free survival (mFOLFOX), *PFS_folfox_shape* shape parameter of progression-free survival (mFOLFOX), *AE* adverse event, *OS_5fu_scale* scale parameter of overall survival (5-FU), *OS_5fu_shape* shape parameter of overall survival (5-FU), *PFS_5fu_scale* scale parameter of progression-free survival (5-FU), *PFS_5fu_shape* shape parameter of progression-free survival (5-FU)

One-way sensitivity analysis indicated that the medication cost of pemigatinib, the utility of the PF state and the PD state, and the parameters of OS were the most crucial parameters influencing the results in both CEAs (Fig. 2a, b). The results of 1000 iterations of the Monte Carlo simulation revealed that compared to either mFOLFOX (Fig. 2c, e) or 5-FU (Fig. 2d, f), pemigatinib was more effective but more expensive, resulting in a 6.5% and 4.6% probability of being cost-effective under a WTP threshold of 3 times the GDP per capita per QALY gained. The value of information analysis showed that the expected value of uncertainty measured by EVPI was NT$43,139/person and NT$23,608/person for mFOLFOX and 5-FU, respectively (Table 2).

### Scenario analysis

**Fig. 3:**
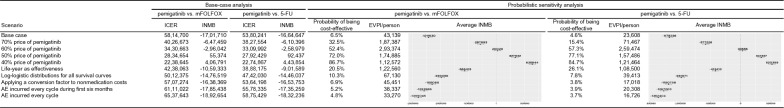
Scenario analysis results: Base-case analysis and probabilistic sensitivity analysis of all scenarios in this study. Costs listed in 2022 NT dollars. *mFOLFOX* a combination of oxaliplatin, folinic acid, and fluorouracil, *5-FU* fluorouracil, *ICER* incremental cost-effectiveness ratio, *INMB* incremental net monetary benefit, *EVPI* expected value of perfect information, *CI* confidence interval, *AE* adverse event

The price scenario showed that pemigatinib achieved cost-effectiveness compared to mFOLFOX or 5-FU when the medication cost of pemigatinib was reduced by 40% (Fig. 3). When the medication cost of pemigatinib was reduced by more than 50%, pemigatinib gained a positive INMB of NT$55,374 and NT$92,437, with a probability of 72.0% and 77.1% of being cost-effective, respectively (Fig. 3). The scenario considering life-years as effectiveness showed that compared to mFOLFOX and 5-FU, pemigatinib was associated with 0.81 and 0.94 increased life-years, resulting in an ICER of NT$4,238,063 per life-year and an ICER of NT$3,888,175 per life-year, with a probability of 20.5% and 26.1% being cost-effective, respectively (Fig. 3). The results of this study were not sensitive to other scenarios, such as using the second-best fit survival models, AE incurred in each cycle, and applying a conversion factor to nonmedication costs. The results just slightly differed from those of the base-case results (probability of pemigatinib being cost-effective compared to mFOLFOX: 4.8–10.3%; probability of pemigatinib being cost-effective compared to 5-FU: 3.7–7.8%; Fig. 3).

## Discussion

Compared to mFOLFOX or 5-FU, pemigatinib was not cost-effective in the base-case analysis when reimbursed at the hypothesized price of NT$17,820 per 13.5 mg. Prior to the middle of April 2023, discussions were onging regarding the listing price of pemigatinib 9 mg/13.5 mg. Taiwan's NHIA was dedicated to cutting the listing price of pemigatinib 13.5 mg to the same as that of pemigatinib 9 mg (hypothesized medication cost: NT$11,880) [13]. When the hypothesized price was reduced by 40% toNT$10,692 per pemigatinib 13.5 mg, the probability of pemigatinib being cost-effective was 52.4% and 57.3% compared to mFOLFOX and 5-FU, respectively. Pemigatinib became cost-effective and was associated with a positive INMB when the hypothesized price was reduced by 50%, approximately to NT$8910 per pemigatinib 13.5 mg. The probability of pemigatinib being cost-effective was 72% in the mFOLFOX comparator case and 77.1% in the 5-FU comparator case. After our completion of this study, Taiwan’s NHIA has announced its decision to reimburse pemigatinib 13.5 mg at NT$12,500, effective since May 2023 [27]. This reimbursement price is 40% higher than the price recommended in our current study.

The current study differs from our previous work in aspects of the target patient population, intervention, and comparators [12]. Our previous work was a guideline-based CEA with the target population of patients with advanced ICC [12]. The intervention was a guideline-based treatment regimen, namely, pemigatinib for patients with *FGFR2* fusions/rearrangements and mFOLFOX (partially covered by NHI) for those without *FGFR2* fusions/rearrangements, while the comparator was 5-FU, the most commonly used second-line regimen for Taiwanese patients with advanced ICC [12]. We used the market price in 2021 for the hypothesized price of pemigatinib 13.5 mg (NT$14,286). Therefore, the previous work was done to answer the following question. Whether the Taiwan NHI should reimburse patients with advanced ICC according to the NCCN guidelines or retain the current reimbursement option only for the 5-FU regimen. The previous work concluded that a 40% or higher price reduction of pemigatinib (NT$8572 for 13.5 mg daily) would make the guideline-based regimen to be cost-effective for all patients with advanced ICC [12].

In contrast, the current study was a trial-based CEA with the target population of ICC patients harboring *FGFR2* fusions/rearrangements. The intervention was pemigatinib and the comparators were mFOLFOX and 5-FU. We used the potentially highest listing price for the hypothesized price (NT$17,820). Therefore, this study was performed to answer the following question. Whether NHI should reimburse the daily use of pemigatinib 13.5 mg in advanced ICC patients with *FGFR2* fusions/rearrangements or keep the current reimbursement option only for the mFOLFOX or 5-FU regimen, based on the trial evidence. The results showed that a price reduction of at least 50% (NT$8910 per 13.5 mg daily) would make the trial-based regimen cost-effective. In summary, current findings of this study better match the need for a reimbursement policy in pricing pemigatinib 13.5 mg  for daily use.

Although this study analyzed the cost-effectiveness of pemigatinib treatment in advanced ICC patients with *FGFR2* fusions/rearrangements, we adopted a similar approach as our previous work and applied most of the parameters. Thus, the current study had the same limitations mentioned previously, including assumptions, data sources, parameter estimations, and the structure of the model [12]. Furthermore, this study was subject to a limitation of selection bias in identifying the target population to estimate parameters of medical care utilization using secondary data from NHI claims data. We were unable to tackle the problem of self-paying circumstances, which may lead to the misclassification of the treatment strategy group.

## Conclusion

As of the completion of this study, Taiwan's NHI had not reached a final decision on the price of pemigatinib 9 mg and 13.5 mg. Our study suggested that a 50% reduction in the hypothesized price for pemigatinib 13.5 mg, NT$8910, would reflect an acceptable cost-effectiveness level. The analytical framework utilized in this study can serve as a reference case for Taiwan's NHIA when evaluating the cost-effectiveness of other FGFR kinase inhibitors in the future.

## Data Availability

The data that support the findings of this study are available from the Ministry of Health and Welfare, Taiwan, but restrictions apply to the availability of these data, which were used under license for the current study and are not publicly available.

## Abbreviations

ICC: Intrahepatic cholangiocarcinoma
CCA: Cholangiocarcinoma
FGFR2: Fibroblast growth factor receptor 2
FDA: Food and Drug Administration
NCCN: National comprehensive cancer network
mFOLFOX: A combination of oxaliplatin, folinic acid, and fluorouracil
NHI: National Health Insurance
5-FU: Fluorouracil
NICE: National Institute for Health and Care Excellence
CADTH: Canada’s Drug and Health Technology Agency
NHIA: National Health Insurance Administration
CEA: Cost-effectiveness analysis
PF: Progression-free
PD: Progressed-disease
PFS: Progression-free survival
OS: Overall survival
ICER: Incremental cost-effectiveness ratio
QALY: Quality-adjusted life-year
INMB: Incremental net monetary benefit
WTP: Willingness-to-pay
GDP: Gross domestic product
DSA: Deterministic sensitivity analysis
PSA: Probabilistic sensitivity analysis
EVPI: Expected value of perfect information
